# Parents' Hesitancy to Vaccinate Their Children Against COVID-19, a Country-Wide Survey

**DOI:** 10.3389/fpubh.2022.755073

**Published:** 2022-04-28

**Authors:** Sultan F. Alhazza, Ali M. Altalhi, Khaled M. Alamri, Saleh S. Alenazi, Bader A. Alqarni, Abdulellah M. Almohaya

**Affiliations:** ^1^Internal Medicine Department, Security Forces Hospital, Ministry of Interior, Riyadh, Saudi Arabia; ^2^Pediatric Cardiology Department, Prince Mohammed Medical City, Ministry of Health, Aljouf, Saudi Arabia; ^3^General Pediatric Department, Prince Sultan Military Medical City, Ministry of Defense, Riyadh, Saudi Arabia; ^4^Pediatrics Department, Ad-Diriyah Hospital, Ministry of Health, Riyadh, Saudi Arabia; ^5^Division of Infectious Diseases, Department of Internal Medicine, Ad-Diriyah Hospital, Ministry of Health, Riyadh, Saudi Arabia

**Keywords:** coronavirus, vaccine, childhood vaccination, hesitancy, Saudi Arabia

## Abstract

**Objective:**

Parents' hesitancy (PH) toward childhood vaccination, including the vaccine of coronavirus disease (COVID-19), is one of the top public health threats. We aim to assess the PH toward children COVID-19 vaccination as compared to PH toward children routine vaccination among the residents of Saudi Arabia.

**Method:**

Before the official approval of children's COVID-19 vaccination in the country, a cross-sectional study using an electronically distributed survey was performed. Responses from parents of children younger than 18 years of age were accepted. The Oxford COVID-19 vaccine hesitancy scale (OC19-VHS) and the routine vaccination hesitancy scale (R-VHS) were used. Parents were classified as hesitant, non-hesitant, and unsure.

**Results:**

Between June 18th−30th, 2021, we included 1,052 parents. More than half of the parents were positive toward the childhood COVID-19 vaccination (63%) while 10% were unsure. Higher parental hesitancy toward children COVID-19 vaccination among mothers, parents younger than 40 years, did not receive COVID-19 nor influenza vaccines, had higher educational levels, and parents who recovered from COVID-19 infection. Hesitancy was mainly driven by the novelty of the vaccines and the fear of serious adverse effects. Compared to the routine vaccination, parents were more hesitant toward COVID-19 vaccination (6 vs. 27%).

**Conclusion:**

Generally, parents in Saudi Arabia were positive toward children's COVID-19 vaccination. Focused education to reassure hesitant parents on the safety of the vaccine is essential to achieve larger vaccination coverage.

## Introduction

Severe Acute Respiratory Syndrome Coronavirus (SARS-CoV-2), as of yet, has caused hundreds of millions of infections and millions of deaths among elderly, adults, and children (1), with mortality risk among hospitalized patients ranging between 10 and 20% including studies from Saudi Arabia (2–4). The urgency to combat such pandemics has led to accelerated competition among scientists and pharmaceutical companies around the globe to create potent and safe vaccines against this disease (5). Such rapid development of these vaccines, as well as other factors, have raised concerns among the general population on safety and efficacy (6). However, these vaccines have been granted emergency use authorizations after they showed large safety and efficacy levels (7, 8).

Hesitancy to vaccination is listed among the top ten threats to global health (9). As per the WHO Strategic Advisory Group of Experts on Immunization (SAGE), vaccine hesitancy is defined as the delay in acceptance or refusal of vaccines despite the availability of vaccine services (10). At our country level, a pre-pandemic study revealed that almost 20% of parents were hesitant with childhood routine vaccination (11), highlighting the magnitude of the vaccination hesitancy in a country that mandates childhood vaccination.

During the pandemic of COVID-19, the crucial role of the COVID-19 vaccine against the pandemic was opposed by a massive infodemic of misinformation and conspiracy theories (12, 13). For many reasons, COVID-19 vaccines evaluation was delayed in the pediatric age group and therefore authorization was granted later in the pandemic. Although COVID-19 had a milder rate of morbidity and mortality among children compared to adults, there are more reasons to vaccinate children against COVID-19. Children might have similar nasopharyngeal viral loads to adults (14), therefore they potentially have a similar risk of viral transmission and rates of secondary infections (15, 16). Moreover, if infected, children are at risk to develop multisystem inflammatory syndrome in children (MIS-C) associated with coronavirus disease 2019 (COVID-19) (17).

The assessment of parents' willingness to provide their children with a vaccine against COVID-19 was performed in different parts of the world, but not in our region. A report by Goldman et al. from six different countries (USA, Canada, Japan, Switzerland, and Spain) where they interviewed parents presenting to emergency departments with their children revealed that only 65% of the parents were willing to vaccinate their children. The likelihood of vaccination was higher if the child was older, free from any comorbid conditions, and in those who were up-to-date in their routine vaccination schedule. The main concerns of hesitant parents were the novelty of the COVID-19 vaccine (18), not being convinced by the vaccine's effectiveness (19), or being unconcerned about COVID severity in childhood (20).

In Saudi Arabia, since July 2021, the BNT162b2 vaccine (Pfizer, USA), mRNA-1273 vaccine (Moderna, USA), ChAdOx1 vaccine (AZD1222, AstraZeneca, UK), and Ad26.COV2.S vaccine (Janssen Inc., USA), remains part of the national vaccination campaign and had shown a high degree of safety (21–23). Until January 2022, 51 million doses of these vaccines have been administered in the kingdom (24). When this study was undertaken, people younger than 18 years of age were denied vaccination as per the Saudi ministry of health protocol.

Thus, we aim to assess the willingness of parents to vaccinate their children against COVID-19 compared to the parent's willingness on the childhood routine vaccination. We also aimed to explore the determinants of the parents' hesitancy on the childhood COVID-19 vaccine.

## Methods

### Study Design and Population

National cross-sectional questionnaire-based survey. Using a pre-designed questionnaire, a link to the survey was distributed electronically to the parents in the general population using social media platforms between June 18 to June 30, 2021. Parents of children aged 18 years or younger who were residents in Saudi Arabia and able to interact with the Arabic questionnaire were eligible to participate. Those respondents who do not complete all the responses were automatically excluded.

### Sample Size Calculation

It is estimated to be 961,392 families in the country (25), for which there are 1,922,784 parents. The sample size was calculated with an estimated 50% response rate, 95% confidence interval, 5% margin of error, and the assumption of normal distribution, the minimum representative sample size is 384. We planned to stop enrollment by the end date of enrollment if more than the minimum enrollment is achieved.

### Study Tool and Questionnaire Development

The questionnaire is divided into three segments: Part-1: Parents and child demographic and past medical history. Part-2: Routine vaccination hesitancy Scale (R-VHS): adopted from the Vaccine hesitancy 9-item scale (26, 27) that asks parents about their views of childhood vaccines. Each item was rated on a 1 (strongly disagree) to 5 (strongly agree) scale. Higher scores indicate greater hesitancy. Part-3: COVID-19 vaccine hesitancy: adopted from the Oxford COVID-19 vaccine hesitancy 7-item scale (OC19-VHS) (28) which has item-specific response options, coded from 1 to 5. Code 4 or 5 was considered a hesitant response, 3 was considered unsure, and code 2 or 1 was considered a positive response, as used by Freeman et al. (28). A ‘Don't know' option was also provided and excluded from the scoring analysis. The scores can range between 7 and 35, with higher scores indicating higher COVID-19 vaccine hesitancy. No modifications to these tools were made. The adopted validated questionnaires were originally written in the English language. Arabic translation and face validation were performed by senior residents fluent in both languages. Next, an English language academician validated the questionnaire with back-translation. A pilot study was carried out among randomly selected volunteers. The Arabic version was modified as per provided feedback in each stage.

### Ethical Considerations

The anonymous survey data were confidentially stored with password-protected security standards. Participants were consented and asked to voluntarily participate and were informed that they, at any time, can terminate their participation and they were told that no incentive to participate. The study was approved by the institutional research board dated June 13, 2021.

### Statistical Analysis

Independent-samples *T*-test was utilized to investigate the difference in the mean score of the COV-Oxford hesitancy scale between study variables. Then, based on the number of hesitant responses on the scale, the respondents were grouped into (1) Hesitant group, when the majority of the individual responses were regarded as hesitant responses (> 60%). The group has a subgroup of strong hesitant if >80%. (2) Non-hesitant group, when the minority of the individual responses were regarded as hesitant responses (< 40%), with a subgroup of strong non-hesitant if no single hesitant response was chosen. (3) Unsure group, when neutral responses account for> 50% of the responses, or there were equal responses between hesitant and non-hesitant responses. Multivariate analysis was performed to explore predictors for parents in the hesitant group. We used the frequency, percentage, mean, and median to present the numerical variables. For comorbid condition definition, we used the United States Center for Disease Control and Prevention definition, conditions that last 1 year or more and require ongoing medical attention or limit activities of daily living, or both. Further statistical analysis was carried out by using the SPSS software (version-23, IBM Corp., Armonk, N.Y., USA). A *p*-value lower than 0.05 was considered statistically significant.

## Results

### Respondents

During the study period, 1,240 visits to the online questionnaire were received, 1,063 individuals confirmed having a child who is under 18 years of age. After excluding 11 incomplete responses, 1,052 participants were included in the analysis. Slightly higher participation was observed from mothers (51.5%, *n* = 542) compared to fathers (48.5%, *n* = 510). The majority of the included parents had Saudi nationality (93.7%) and were from healthy medical backgrounds (82.2%). Most of the parents have reported their level of education as a University degree (62.5%) or postgraduate studies (24.2%), while the remaining participants had a secondary school degree (8.1%) or others. The top sector of job among the parents in this cohort was the education sector (27.4%), followed by the healthcare sector (20.3%), administrative jobs sectors (17.3%), housewives (15.6%), or others. Half of the included parents have reported receipt of the influenza vaccine (51.2%) over the last year. Most of the respondents themselves had already received at least one dose of the COVID-19 vaccine (*n* = 854/1052, 81.1%) and only the minority had a history of laboratory-confirmed COVID-19 (*n* = 202/1052, 19.2%). Nevertheless, among those who yet did not receive COVID-19 vaccine (18.8%, *n* = 198/1052), ten percent have no intention to vaccinate themselves soon (*n* = 105/1052). When parents were asked about the burden of COVID-19, at least one in each three had a close relative or friend who died of COVID-19, while one in each two knows a child who suffered from laboratory-confirmed COVID-19. Most of the parents (77.7%) have disclosed that their decision of vaccinating their kids will not change according to the child's age, gender, or comorbid conditions. Each parent had a median of four children, who were predominantly boys (41.4%), predominantly girls (37.4%), or have an equal distribution between the two genders (21.2%). Only 13.4% of the parents have disclosed a history of laboratory-confirmed COVID-19 among their children. The majority of parents have reported a full immunization status for their kids at least until 2 years of age (95.9%). There was a lower rate of children with comorbid disease (8.5%), history of self-reported allergy that at some point required medical attention (13.7%), or serious adverse effects following any vaccine (4.8%) (Table 1).

**Table 1:** Basic characteristics (*n* = 1,052).

**Parameters**		**Count**	**%**
Parent	Mother	542	51.5%
	Father	510	48.5%
Parent's age	< = 40 years	602	57.2%
	> 40 years	450	42.8%
Parent's nationality	Saudi	986	93.7%
	Non-Saudi	66	6.3%
Parent's education	University degree	657	62.5%
	Post-graduate studies	255	24.2%
	Secondary school	85	8.1%
	Pre-secondary school	32	3.0%
	Other	23	2.2%
Parent's Job	Education sector	288	27.4%
	Healthcare sector	214	20.3%
	Administration sector	182	17.3%
	Housewife	164	15.6%
	Military sector	107	10.2%
	Other	51	4.8%
	Private Business	37	3.5%
	Unemployed	9	0.9%
Parent's past history	Have comorbid condition	187	17.8%
	Received Flu vaccine last year	539	51.2%
	Diagnosed with COVID-19	202	19.2%
Parent's COVID-19 vaccine	Yes and fully convinced	236	22.4%
	Yes, but not fully convinced	618	58.7%
	No, but I will do that.	93	8.8%
	No, and will never do	105	10.0%
Current number of children	1 Child	31	2.9%
	2 Children	88	8.4%
	3 Children	160	15.2%
	4 Children	254	24.1%
	5 Children	306	29.1%
	6 or more children	213	20.2%
Your child/children's gender	Equal girls and boys	223	21.2%
	Most are boys	196	18.6%
	Most are girls	197	18.7%
	All boys	240	22.8%
	All girls	196	18.6%
Relatives with COVID-19	Knows relative died of COVID-19	346	32.9%
	Knows relative child diagnosed with COVID-19	544	51.7%
	Have comorbid conditions	89	8.5%
	History of allergy required medical evaluation	144	13.7%
	Up-to-date routine vaccination	1,009	95.9%
	History of severe adverse effects after vaccine	50	4.8%
	Had COVID-19	141	13.4%
Will your decision change based on the child's age, gender, or comorbid condition?	No	817	77.7%
	The older	135	12.8%
	Not sure	83	7.9%
	The presence or absence of comorbidities	33	3.1%
	The males	2	0.2%

### Parents' Hesitancy on the Childhood COVID-19 Vaccine

**Figure 1:**
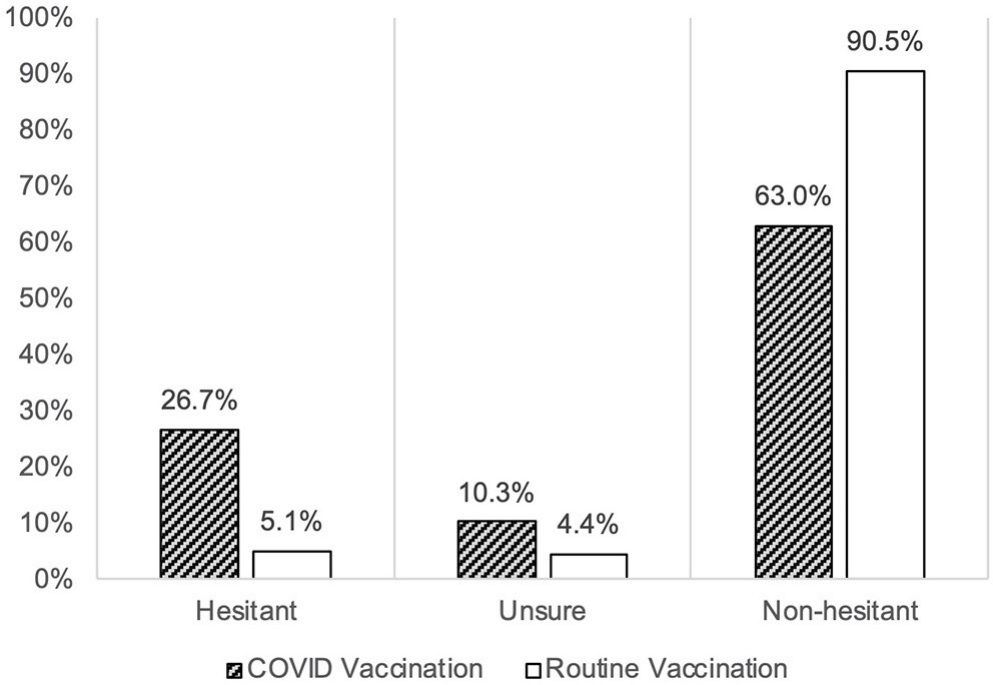
The parental hesitancy toward childhood COVID-19 vaccination (Oxford COVID-19 Vaccine Scale) as compared to the hesitancy toward routine childhood vaccination (VHS scale), among 1,052 parents in Saudi Arabia between June 18 to June 30, 2021.

Using the Oxford COVID-19 vaccine hesitancy score (OC19-VHS), we found that the mean score of hesitancy toward COVID-19 vaccination was 18 out of 35 points. Most of the parents fell in the non-hesitant group (63%, *n* = 663/1052), while the remaining were either in the hesitant group (27%, *n* = 281/1052) or unsure about their decision (10%, *n* = 108/1052) (Figure 1). Furthermore, 6% were considered a strongly hesitant subgroup. On the other side, 48% of the parents were among the strongly non-hesitant group.

### Comparison to the Childhood Routine Vaccination

**Figure 2:**
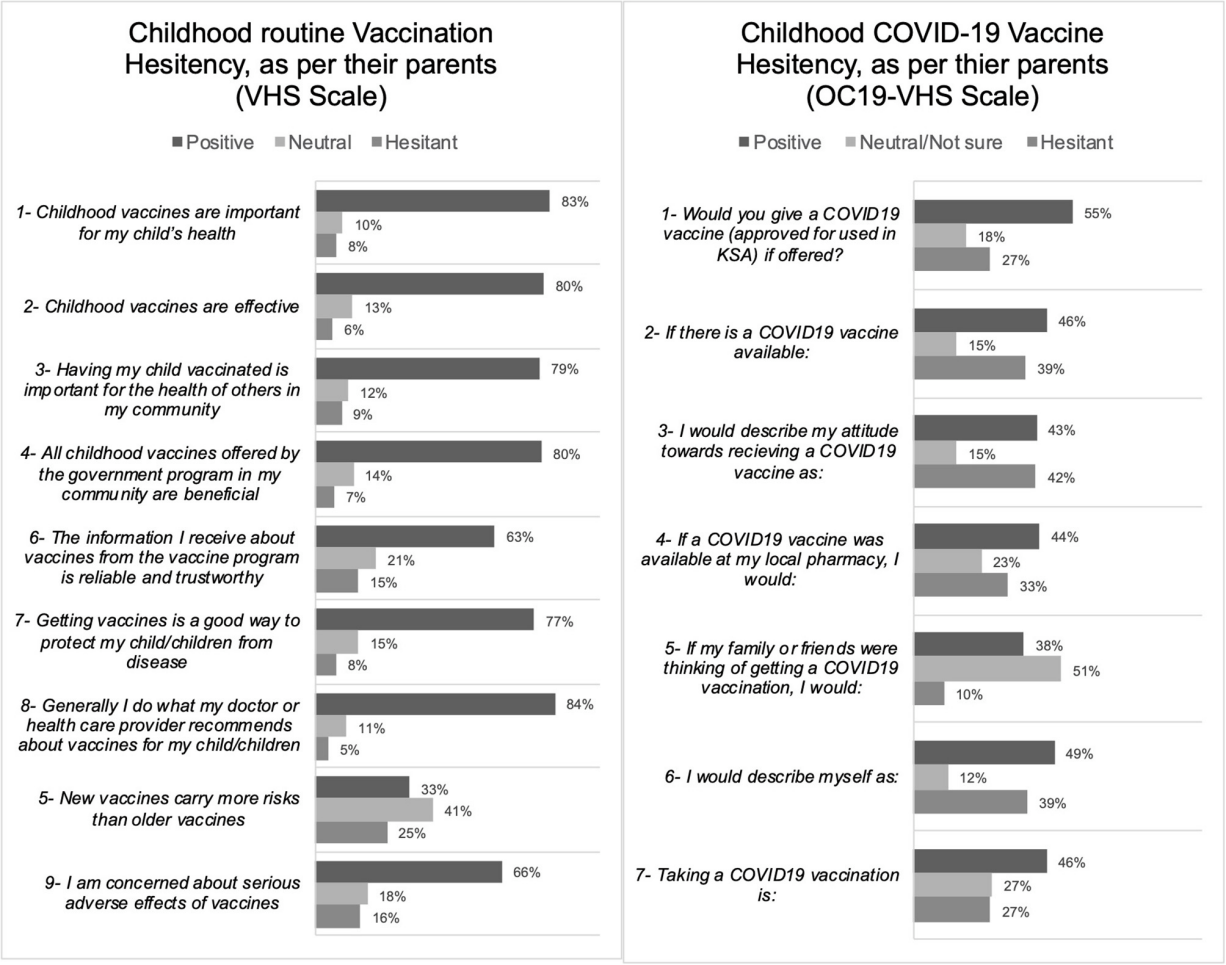
The frequencies of endorsement of each item are categorized into hesitant (Category 4 and 5), positive (Category 1 and 2), or neutral (*n* = 1,052).

The mean hesitancy score among parents toward childhood COVID-19 vaccination was found higher (18/35 points (51%) in OC19-VHS) compared to the hesitancy toward the routine childhood vaccination [19.5/45 points (44%) in VHS]. The major drive for such difference was the higher proportion of the hesitant group against the childhood COVID-19 vaccine (27%) compared to the hesitant group on the childhood routine vaccination (6%). Additionally, the proportion of the unsure group was higher in the childhood COVID-19 vaccination (10%) compared to the unsure group in the childhood routine vaccination (4%) (Figure 1). Detailed results on the responses to both scales are presented in Figure 2.

### Determinant of the Parent's Hesitancy on the Childhood COVID-19 Vaccination

We investigated the determinants of the parent's hesitancy on the childhood COVID-19 vaccination and also the childhood routine vaccination. In both types of vaccinations, we found significantly higher hesitancy mean scores in mothers (compared to fathers), parents who are younger than 41 years of age (compared to older than 40 years of age), parents who never received influenza or COVID-19 vaccines (compared to those who did), and those whom their children had a history of allergy after any previous vaccine (compared to those who did not). Further, the unique determinants of the hesitancy on the childhood COVID-19 vaccination alone (but not childhood routine vaccination) were those parents with an education level of a University degree or above (compared to a lower education level) and parents with a history of COVID-19 diagnosis (compared to those never diagnosed with COVID-19). On the other side, the unique determinants of the hesitancy on the routine childhood vaccinations alone (but not the childhood COVID-19 vaccination) were parents with lower than 4 children (compared to those with more than 4 children), parents with children who had a history of COVID-19 diagnosis (compared to those who did not), parents with children who did not complete the routine childhood vaccination schedule (compared to those who did) (Table 2).

**Table 2:** Association and means comparisons between the study variables for COV-Oxford 7-item score (*n* = 1,052), higher scoring in the scales indicate higher hesitancy.

**Study parameters**			**Total COV-Oxford 7-item score (out of 35)**	
			**Mean**	**Sig. (2-tailed)**
Parent	Relation	Mother	18.9	<0.001*
		Father	16.9	
	Age	< = 40 years	19.1	<0.001*
		> 40 years	16.4	
	Nationality	Non-Saudi	17.8	0.845
		Saudi	18	
	Education	University or above	18.3	0.005*
		Lower than University	16	
	Past history	Comorbid condition	16.8	0.045
		Previously healthy	18.2	
	Previous COVID-19	Yes	19.7	0.003*
		No	17.6	
	Received Flu vaccine last year	Received	15.7	<0.001*
		Not Received	20.4	
	COVID vaccine	Received	16.4	<0.001*
		Not Received	24.7	
	Knows relative died of COVID-19	Yes	17.5	0.239
		No	18.2	
	Knows relative child diagnosed with COVID-19	Yes	18.2	0.487
		No	17.8	
Children	Gender	Predominant girls	18	0.764
		Predominant boys	17.5	
		Equal	18.8	
	Number of children	< =4	18.4	0.094
		>4	17.5	
	Past history	Comorbid condition	16.2	0.059
		Previously healthy	18.1	
	Previous COVID-19	Yes	18.8	0.244
		No	17.8	
	Completed childhood vaccination	Yes	17.9	0.665
		No	18.6	
	Allergy history	Yes	19.2	0.076
		No	17.8	
	History of severe adverse events following vaccine	Yes	22.2	0.001*
		No	17.8	

Multivariate regression analysis was performed to determine independent predictors for hesitant or strongly hesitant parents on childhood COVID vaccination, and resulted in a significant association with hesitancy toward routine childhood vaccination (adjusted odds ratio (aOR) 8.08 [95% CI 3.821–17.086)]), COVID-19 unvaccinated parents (aOR 3.398, 95% CI 2.342–4.93), flu unvaccinated parents (aOR 1.690, 95% CI 1.235–2.313), or parents with University education and higher (aOR 2.016, 95% CI 1.217–3.339) (Table 3).

**Table 3:** Multivariate analysis on predictors of hesitant or strongly hesitant position in parents toward childhood COVID-19 vaccination based on Oxford COVID-19 vaccine hesitancy scale (OC19-VHS) (28).

**Variables**	**B**	**S.E**.	**Wald**	**Sig**.	**Exp(B) (95% CI)**
Parent education, University, or above	0.701	0.257	7.421	0.006*	2.016 (1.217–3.339)
Parent with no history of flu vaccination	−0.525	0.160	10.739	0.001*	1.690 (1.235–2.313)
Parent with no COVID vaccination	1.223	0.190	41.512	0.000*	3.398 (2.342–4.930)
Hesitancy toward routine childhood vaccination (hesitant or strongly hesitant as per VHD scale)	2.089	0.382	29.905	0.000*	8.080 (3.821–17.086)

## Discussion

In this study, we found a quarter of parents in Saudi Arabia were hesitant toward children's COVID-19 vaccination. In the same cohort, such hesitancy was found three times more than the hesitancy toward routine childhood vaccination, especially among mothers, parents who were younger the 40 years of age, and parents of children with allergies.

Children's vaccination has been successful in preventing many infectious diseases and was considered more effective than elderly vaccination to achieve community-wide prevention of certain infectious diseases (29). In COVID-19, children's vaccination does not only protect them but might provide an indirect advantage in protecting the older unvaccinated people (30). Globally, early in the pandemic or recent data both have revealed an elevated parental hesitancy toward the children's COVID-19 vaccination (18, 31, 32). Regionally, a recent study from Kuwait showed substantially higher parental rejection (55.8%) for children's COVID-19 vaccination (33). In our study, however, the majority of the participants were not hesitant toward the COVID-19 or routine childhood vaccination. COVID-19 vaccine-hesitant respondents in the current study were concerned that it is a new vaccine of unknown adverse events and do not trust its effectiveness, which replicates similar local studies' findings (34, 35). Some of them have expressed a lack of trust in the information provided by the official agencies. The fact that the hesitancy is more when it comes to COVID-19 compared to the routine childhood vaccination disfavor anti-vaccination ideology, but rather a lack of enough public awareness on the safety and efficacy among the pediatric age group. An additional consideration is the timing of the survey to the status of the pandemic. For example, a surge in the acceptance of enrollment in the COVID-19 trial was noted during the stages of higher disease spread (36), thus, a relatively controlled COVID-19 status in Saudi Arabia during the study period of June 2021 (37), might have affect intention of vaccination. Without a doubt, the massive spread of misinformation during the pandemic of COVID-19 has caused significant challenges to public health strategies, especially in the era of social media, and is still a hard task to counteract (12).

Sociodemographic determinants of a higher parental hesitancy toward children COVID-19 vaccination in this study were replicated from previous studies. First, parents who were mothers were found more hesitant, compared to fathers (18, 38, 39). Further exploration in our cohort revealed that the mothers had higher rejection of flu vaccination (57 vs. 41%, *p* < 0.001) and self-vaccination against COVID-19 (25.1 vs. 12.1%, *p* < 0.001) compared to the fathers. Although this needs further investigations including special concerns on pregnancy outcome, but also could alarm early antivaccination behavior among mothers in Saudi Arabia. The other consideration is that mothers in our cohort know more children with a history of COVID-19 diagnosis (61 vs. 42%, *p* < 0.001) than fathers, which probably has decreased their anxiety toward COVID-19 in children. Second, parents who were younger than 40 years of age or those with higher educational backgrounds were more hesitant toward children's COVID-19 vaccination, as seen in previous studies (18, 31, 35, 38, 40). Previous studies found these groups were more likely to have selected sources of information and they base their decision on a critical-thinking attitude (40). Thus, public health messaging should consider such targets of the population with the appropriate mode of messaging. It is worth mentioning that such parent gender and age differences were not found significant after the multivariate regression analysis.

Consistent with previous studies (18, 38), vaccinated parents would be more willing to vaccinate their children, while those who had COVID-19 in a mild form would be more hesitant. This alerts toward the effect of parental self-experience on children's healthcare decisions.

This study has several limitations. We did not evaluate the impact of family income and the impact of the child's age on the parent's decision of childhood COVID vaccination. Although the sample size was deemed adequate statistically, the recruited sample was at risk of selection bias to those who use social media as the recruitment tool which might underestimate vaccine hesitancy among other populations. Future studies investigating the beliefs and cultural influences of parental hesitancy are recommended.

## Conclusion

Only one in every four parents in Saudi Arabia was hesitant toward children's COVID-19 vaccination, which is similar to the rate reported worldwide. The focus of public health campaigns on the hesitant population, including mothers, younger parents, highly educated parents, and parents with a history of COVID-19 infection might help to decrease parents' hesitancy toward children's COVID-19 vaccination aiming to achieve further control of COVID-19 in the community.

## Data Availability Statement

The datasets presented in this article are not readily available because it could compromise the privacy of research participants. Requests to access the datasets should be directed to AAlm, almohayaam@gmail.com.

## Ethics Statement

The studies involving human participants were reviewed and approved by the Institutional Research Board (KFMC/KACST-IRB: H-01-R-012, OHRP/NIH: IRB00010471) dated June 13, 2021. The patients/participants provided their written informed consent to participate in this study.

## Author Contributions

AAlm, SFA, and BA: proposal preparation. AAlm, SFA, AAlt, and KA: manuscript writing. All authors contributed in study concept and methodology, data collection, analysis, interpretation, manuscript review, and final approval.

## Conflict of Interest

The authors declare that the research was conducted in the absence of any commercial or financial relationships that could be construed as a potential conflict of interest.

## Publisher's Note

All claims expressed in this article are solely those of the authors and do not necessarily represent those of their affiliated organizations, or those of the publisher, the editors and the reviewers. Any product that may be evaluated in this article, or claim that may be made by its manufacturer, is not guaranteed or endorsed by the publisher.
